# MinION: A Novel Tool for Predicting Drug Hypersensitivity?

**DOI:** 10.3389/fphar.2016.00156

**Published:** 2016-06-14

**Authors:** Eng Wee Chua, Pei Yuen Ng

**Affiliations:** Faculty of Pharmacy, National University of MalaysiaKuala Lumpur, Malaysia

**Keywords:** MinION, nanopore sequencing, drug hypersensitivity, sequence-based HLA typing, bioinformatics analysis

## Abstract

The launch of the MinION Access Program has caused much activity within the scientific community. MinION represents a keenly anticipated, novel addition to the current melange of commercial sequencers. Driven by the nanopore sequencing mechanism that requires minimal sample manipulation, the device is capable of generating long sequence reads in sizes (up to or exceeding 50 kb) that surpass those of all other platforms. One notable advantage of this feature is that long-range haplotypes can be more accurately resolved; such advantage is particularly pertinent to the genotyping of complex loci such as genes encoding the human leukocyte antigens, which are pivotal determinants of drug hypersensitivity. With this timely, albeit brief, review, we set out to examine the applications on which MinION has been tested thus far, the bioinformatics workflow tailored to the unique characteristics of its extended sequence reads, the device’s potential utility in the detection of genetic markers for drug hypersensitivity, and how it may eventually evolve to become fit for diagnostic purposes in the clinical setting.

## Introduction

The launch of the MinION Access Program by Oxford Nanopore Technologies (ONT), a UK-based company specializing in nanopore sequencing, has caused much activity within the scientific community. The device is a miniature, third-generation sequencer in which 512 nanopores are housed and responsible for sensing single-stranded DNAs. With steadily improving sequencing accuracy, MinION has been a much welcome addition to the melange of tools deployed for diagnosing inherited drug hypersensitivity. The compact measurements of the device confer it such a degree of portability that is unsurpassable by other platforms. There has even been speculation that MinION could be transported to Mars and used to probe the existence of alien life forms (Check Hayden, 2015). Thus far, the use of MinION has been directed largely toward DNA sequencing; however, a broader range of MinION applications for RNA, microRNA, and protein analysis are being explored. A scaled-up version of MinION, which is composed of 48 flow cells and designated PromethION, has also been made available via another program, granting the participants early access to the platform (Karow, 2015).

## MinION: History and Applications

The characteristics of ion channels as nanopores for DNA molecule detection have been extensively investigated since two decades ago (Kasianowicz et al., 1996; Howorka et al., 2001). When purified, single-stranded DNAs are passed through an array of pores embedded onto a membrane, characteristic current patterns that reflect the identities of DNA bases are produced (Clarke et al., 2009; Ip et al., 2015). Various improvements in the experimental setup have increased the accuracy of base-reading; these have included modification of the structure of α-hemolysin channels, addition of bulky cyclodextrin molecules to reduce the speed of DNAs translocating through the pores, introduction of hairpin polynucleotides to connect unzipped, double-stranded DNAs, and adjustment of salt concentrations in the buffer to alter the voltage across the membrane (Clarke et al., 2009; Brown et al., 2012). These changes have ultimately led to the release of MinION. Compared with existing second-generation sequencers such as MiSeq and Ion Torrent that sequence up to 400 bp, MinION is deemed ideal for DNA sequencing as it can generate long reads up to or exceeding 50 kb (Jain et al., 2015). Moreover, it does not require pre-amplification of the sample, which removes potential bias in the data that could be introduced by polymerase chain reactions (PCRs).

**FIGURE 1:**
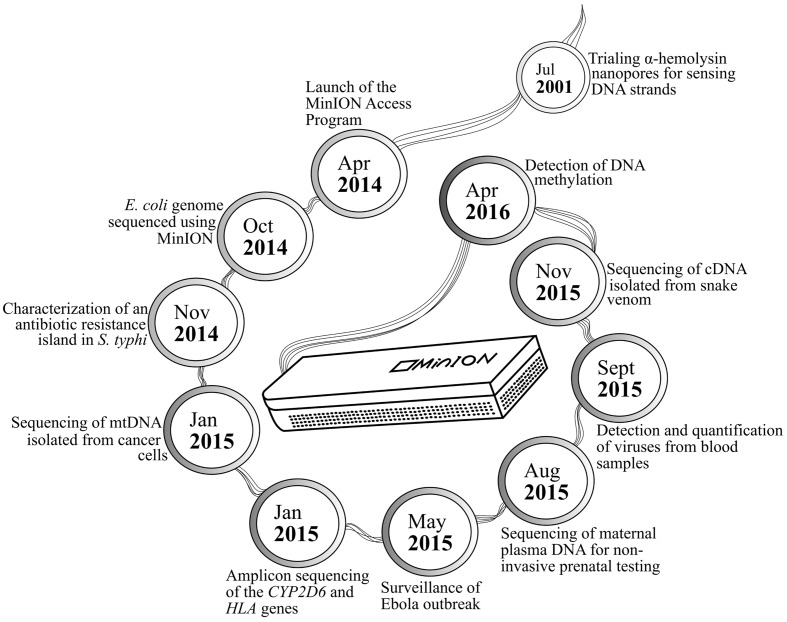
**MinION applications presented in chronological order**.

MinION has been tested in diverse applications, ranging from early work on bacterial sequencing and identification, to recent discovery of its ability to detect pathogens in human plasma and that to distinguish methylated DNA bases from their unmethylated counterparts (**Figure 1**). Extended sequences generated by MinION have been used to supplement better quality MiSeq data in constructing the genomes of *Bacteroides fragilis* and *Saccharomyces cerevisiae* (Goodwin et al., 2015; Risse et al., 2015). Further enhancement of bioinformatics workflow has allowed *de novo* genome assembly for the *Escherichia coli* K-12 MG1655 strain, whereby 98.4% nucleotide accuracy was noted across the 4.6-mb reconstructed genome (Loman et al., 2015). Such high-quality data output from the device has later been translated into differentiating three closely related poxviruses, namely cowpox, vaccinia-MVA, and vaccinia-Lister (Kilianski et al., 2015). The portability of MinION and relatively rapid sample preparation and data generation associated with the sequencer have positioned it to be a useful tool in the recent Ebola epidemic, wherein it was used on-site for monitoring the evolution of Ebola virus in a series of clinical samples. Similar principles of viral identification from human blood have also been applied to sequencing chikungunya and hepatitis C viruses (Greninger et al., 2015; Quick et al., 2016).

In the detection of DNA methylation patterns in human samples, the subtle differences in the electrical signals produced by methylated cytosine bases could also be picked up by MinION (Simpson et al., 2016). This is an exciting breakthrough as the conventional identification of these molecular modifications requires a more complex and specialized sample preparation approach, such as methylated DNA immunoprecipitation followed by sequencing, or bisulfite genomic sequencing (Frommer et al., 1992; Corley et al., 2015). Bisulfite sequencing, the gold standard for detecting DNA methylation, indirectly allows differentiation of methylated cytosines via selective conversion of unmethylated cytosines to uracils, which are then detected as thymine *post*-PCR (Li and Tollefsbol, 2011). In contrast, the MinION-based workflow is much simpler: a statistical model has been developed and optimized for direct identification of 5-methylcytosine from samples prepared by ONT’s standard protocol (Simpson et al., 2016). Though a direct causal relationship between drug hypersensitivity and DNA methylation has not been observed, the epigenetic phenomenon has been proven to mediate other processes such as disease pathogenesis and the effectiveness of a number of drugs (Beyrouthy et al., 2009; Anier et al., 2010).

## Processing of Nanopore Sequencing Data

Central to the processing of nanopore sequencing data is the hidden Markov model (Eddy, 2004), which has been applied to various stages of sequence analysis from base-calling, fine-tuning alignment, to variant discovery (Jain et al., 2015; Szalay and Golovchenko, 2015). For base-calling, this model yields statistical deductions about the underlying DNA sequences (hidden state) based on a series of *emitted* observations. The successive ionic perturbations (events) caused by 6-mer DNAs (or 5-mer DNAs for now-obsolete workflows) that are drawn through the nanopores are rendered into DNA sequences based on known pairs of DNA sextets and corresponding current values (Timp et al., 2012; Szalay and Golovchenko, 2015). A major obstacle to this approach is that the current levels for all possible 6-mer combinations comprise a continuum of electrical signals rather than segregate into discrete patterns that could be unambiguously interpreted. To tease out these ionic signatures, additional clues are gained from neighboring sequences (Timp et al., 2012). For instance, suppose we were to infer a 6-mer DNA from an electrical signal, which indicated there were two possibilities: TACGTA and TACGTT. We knew that, on most occasions, the preceding sequence, ATACGT, was likely to transition to TACGTA; thus TACGTA should have been the sequence motif from which the signal had originated (Timp et al., 2012). Though this example is rather simplistic, it serves to illustrate the effectiveness of the Markov model. The emission and transition probabilities can be derived from datasets that are used to train the model.

Similarly, for sequence alignment, *maximum likelihood estimates* can be computed for all nanopore sequencing error types within a Markov network, i.e., insertions, deletions, and substitutions. These estimates are then used to ascertain whether a reference-discordant read is indeed misaligned. For instance, as A–T or T–A miscalls are unlikely, sequence alignments containing these mismatches may well have been incorrectly placed. Alternately, the aligner may not be at fault and the discrepancies may have arisen from inherent DNA variation (Jain et al., 2015). This strategy has been extrapolated to enhance alignments generated by existing aligners and subsequently detect variants deliberately introduced into a phage genome reference (Jain et al., 2015).

**FIGURE 2:**
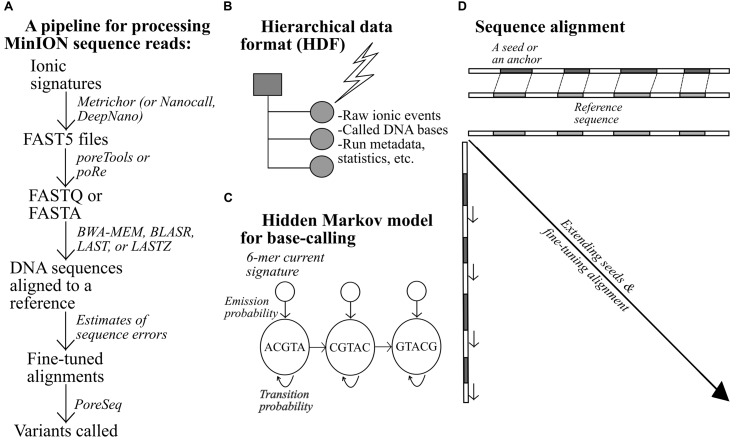
**An example of a pipeline for the processing of MinION data (A), (B–D) alongside additional notes for relevant bioinformatics-related concepts**.

An example of a bioinformatics pipeline for MinION-generated data is shown in **Figure 2**. Raw electrical signals can be base-called by a tool provided by ONT, Metrichor, or other open-source software (Boža et al., 2016; David et al., 2016). DNA sequences produced from the nanopores are stored in the FAST5 format, alongside other types of data such as the run statistics. All data are stratified and placed within predefined categories. Pre-alignment processing typically requires extraction of FASTA or FASTQ sequences from the FAST5 files, using Poretools (Python-based; Loman and Quinlan, 2014) or poRe (written in R; Watson et al., 2015). A number of long-read aligners have been tested thus far on nanopore sequences, including BLASR, BWA-MEM, LASTZ, and LAST. One of the challenges unique to long-read alignment lies in quickly finding short matches, termed *seeds* or *anchors*, between two sequences during a preliminary round of alignment (Li and Durbin, 2009; Chaisson and Tesler, 2012; Li, 2013). With longer sequences, the numbers of possible matches and mismatches are larger; hence, the operation of the aligners would be affected, to a greater extent, by the efficiency of their *seed-and-extend* algorithm.

BLASR and BWA-MEM first invoke *Burrow-Wheelers transform* to create easily searchable, sorted strings (index) of a reference genome to facilitate initial side-by-side check that pinpoints short *exact* matches, which are subsequently extended or refined to form longer DNA stretches (Chaisson and Tesler, 2012; Li, 2013). LASTZ resembles BLASR and BWA-MEM but with two principal variations. First, the aligner employs a different indexing mechanism and dissects the reference sequence into equally sized, overlapping segments to ease sequence comparison. Second, it does not require perfect similarity for qualifying a short match as an acceptable seed; some degree of discrepancy is permitted (Harris, 2007). LAST differs from LASTZ in that it can resolve repeat-rich sequences more successfully (Kiełbasa et al., 2011). When aligned, the sequences can be further scrutinized using NanoOK, which works out the read-length distribution, the occurrence of k-mers, the depths of coverage across targets, and other relevant statistics (Leggett et al., 2015). With accurate alignments, high-confidence DNA variant calls can then be generated.

## Pharmacogenetics: A Classic Case of Abacavir Hypersensitivity

Abacavir, a nucleoside reverse-transcriptase inhibitor used to treat HIV-1 infection, causes a potentially fatal hypersensitivity reaction, to which Caucasians are notably susceptible, in 4–9% of individuals exposed to the drug (Hetherington et al., 2002; Mallal et al., 2002; Symonds et al., 2002; Martin et al., 2004). Abacavir-induced hypersensitivity remains, to date, one of the few rewarding examples of pharmacogenetics-guided therapy. Genetic predisposition, specifically the presence of the *HLA-B^∗^57:01* allele (human leukocyte antigen), is a strong predictor of abacavir hypersensitivity. The association, first reported in a Western Australian population (Mallal et al., 2002), has since been replicated in many other studies (Hetherington et al., 2002; Hughes et al., 2004a; Rauch et al., 2006; Rodriguez-Novoa et al., 2007; Waters et al., 2007; Zucman et al., 2007; Mallal et al., 2008). The *HLA-B^∗^57:01* allele has a high degree of penetrance; the pooled odds ratio from three studies was computed to be 29. Nevertheless, not all *HLA-B^∗^57:01* carriers would be hypersensitive toward abacavir; of every ten individuals harboring the allele, only five would indeed develop a reaction (Hetherington et al., 2002; Mallal et al., 2002; Hughes et al., 2004a). It is likely that other gene loci or pathways, particularly those implicated in the conversion of abacavir into allergenic metabolites, have also contributed to the pathogenesis of abacavir hypersensitivity (Martin et al., 2004, 2012).

Screening for *HLA-B^∗^57:01* carriage, and using this information to preclude susceptible individuals from receiving abacavir, substantially reduced the incidence of abacavir hypersensitivity, hence relieving the costs that would have been incurred by the management of these reactions (Hughes et al., 2004a; Schackman et al., 2008). The decrease in the incidence of hypersensitivity reactions ranged from two-fold (Waters et al., 2007; Mallal et al., 2008) or four-fold (Rauch et al., 2006) to complete eradication (Zucman et al., 2007). The cost-efficiency of *HLA-B^∗^57:01* testing is ethnicity-dependent, being largest in an all-Caucasian or a predominantly Caucasian population. Individuals of other ethnic origins, such as Africans (Hetherington et al., 2002; Hughes et al., 2004b) and Taiwanese (Sun et al., 2007), derive little benefit from such a discriminative strategy. The risk of developing abacavir hypersensitivity is considerably lower in both populations (Symonds et al., 2002; Sun et al., 2007). For instance, the incidence of abacavir-induced hypersensitivity is only 0.9% among Taiwanese individuals, coinciding with their equally rare carriage of the *HLA-B^∗^57:01* allele (0.3% versus about 8% in Whites; Rauch et al., 2006; Sun et al., 2007). Despite the obvious influence of individual ethnic backgrounds, the Clinical Pharmacogenetics Implementation Consortium has recommended *HLA-B^∗^57:01* screening for all patients before they are given abacavir (Martin et al., 2012, 2014). Distinguishing *HLA* alleles could be challenging, as these alleles may vary at only a few positions within their second and third exons (Robinson et al., 2015).

### Use of the MinION Device to Genotype *HLA* Alleles

Several test options exist for *HLA-B^∗^57:01* screening, with sequence-based methods being most technically complex and deemed unsuited for routine use (Martin et al., 2012). These methods often rely on PCRs to enrich the desired *HLA* regions (Carapito et al., 2016). In a bespoke, PCR-based pipeline (Ammar et al., 2015), the *HLA-A* and *HLA-B* regions were amplified in two long-range PCRs and the resultant products (~4 kb each) were sequenced on MinION. The accuracy of the nanopore reads was expectedly low, with the proportion of reference-discordant bases nearing 30%. The authors surmised that this could have adversely affected the genotyping results: the two samples trialed using this method were both erroneously typed. The possibility of the *HLA* haplotypes being obscured by PCR recombination or *chimerism* was alluded to but not thoroughly discussed (Ammar et al., 2015; Laver et al., 2016).

Cross-over extension or template switching is a well-known PCR artifact whereby incompletely synthesized PCR products (mega-primers) anneal to new templates giving rise to chimeric alleles (Odelberg et al., 1995). Recombined alleles would confound haplotype interpretation and it is noteworthy that such errors are clinically significant and could diminish the efficacy of MinION-centric approaches for *HLA* genotyping. For instance, in the case of characterizing the *BCR-ABL1* gene in chronic myeloid leukemia, the occurrence of PCR recombination was found to create artificial compound *BCR-ABL1* mutations, potentially misguiding anti-cancer therapy. After two rounds of PCR, totalling 80 cycles, nearly 50% of the amplicons were noted to be chimeric (Parker et al., 2014).

Several alterations could be made to the PCR protocol to attenuate template switching. For instance, the extension time could be prolonged to ensure complete synthesis of PCR products in each cycle; or the number of PCR cycles curtailed to reduce the probability of PCR recombination (McDonald et al., 2002; Laver et al., 2016). Cross-over events are more likely to occur near the end of PCR cycling or following numerous rounds of extension, during which the synthesized products are most concentrated. Alternatively, a different mechanism of enrichment could be adopted to obviate the need for these changes. Short, complementary oligonucleotides, acting as *probes*, can efficiently *capture* target *HLA* regions from a pool of genomic DNAs that have been trimmed to a pre-defined size range (Wittig et al., 2015). For MinION, it may be necessary to opt for longer probes (Karamitros and Magiorkinis, 2015) and a much less severe DNA fragmentation protocol, in order to preserve the capacity of the device to produce very long reads for the assembly of large-scale haplotypes.

Presently, a more pressing concern over MinION is perhaps its low base-calling accuracy; the error rate has frequently been estimated to exceed 10% (Check Hayden, 2015). Previous attempts at overcoming this problem have tackled chiefly two aspects of the sequencing workflow: conversion of input DNAs into sequenceable templates; and translation of ionic events into DNA bases. The first tactic derives benefit from redundant sequencing in that the input DNA is circularized and amplified in a segmental manner (rolling circle amplification) to generate tandem copies (≥6) of a segment for consensus sequence determination (Li et al., 2016). This has resulted in greatly increased read accuracy (>97%) that approach those attainable by the second-generation platforms (Quail et al., 2012). However, the cost of such improved accuracy is a reduced *net* output from MinION; in other words, fewer bases are ultimately emitted per run. The second tactic is based on statistical learning enhanced by the rapidly expanding MinION datasets that are openly accessible. This has enabled mature hidden Markov models to be built for base-calling, establishing more precise event-to-sequence patterns. The construction of a sophisticated artificial neural network for DNA sequence deduction has also been suggested as a solution to the conundrum (Jain et al., 2015; Boža et al., 2016; David et al., 2016). Currently, it is uncertain which of the two strategies is superior: modified template preparation which adds some degree of complexity, or better trained *in silico* algorithms?

The relatively time-consuming nature of sequence-based *HLA* typing constitutes another limitation that must be overcome to enable routine use of the technique. For instance, it may not be uncommon for a MinION sequencing run to take one day, though it is possible to analyze the data prior to conclusion of the run. The requirement for long-range PCR amplification to isolate *HLA* genes could easily lengthen the procedure to span two days. For the synthesis of long amplicons, protracted elongation time is an inevitable bottleneck; 30–60 s are typically needed for the polymerization of ~1000 nucleotides. A probe-based protocol may entail an even longer time of 3–4 days; but it offers the advantage of eradicating PCR chimerism by needing only 18 cycles of amplification for the enrichment of captured fragments (Karamitros and Magiorkinis, 2015; Laver et al., 2016). On the other hand, an expedited long PCR protocol may be formulated from the following ingredients: whole-blood PCR which eliminates the need for DNA extraction (Mercier et al., 1990), ultra-fast PCR empowered by prompt temperature switches (Wheeler et al., 2011), and highly processive *Taq* polymerases capable of incorporating nucleotides at a faster rate (Böhlke et al., 2000).

## Conclusion

As we have pointed out, several issues warrant deliberation before MinION-based *HLA* typing could be considered for clinical use. The unsatisfactory data accuracy is still an unresolved issue. A modified preparatory protocol that unifies sequence information from tandem copies of a DNA segment has been shown to augment base-calling accuracy (Li et al., 2016). It may be worthwhile to compare the performance of this technique with other *in silico* approaches. The turnaround time for sequence-based tests needs to be drastically shortened; also, the testing process should be complemented by a streamlined mechanism for data analysis, interpretation, and reporting. Spartan RX, a panel indicated for the identification of CYP2C19 poor metabolizers, can generate the required result within 1 h of sample acquisition (Spartan Bioscience Inc., 2016). Above all, the actual utility of sequence-based tests begs the question as to whether the level of *HLA* genotype resolution achieved by other quicker and less laborious methods, such as allele-specific PCR, is already sufficient in the clinical setting (Martin et al., 2012). Despite these uncertainties, the diverse utility of MinION is evident in the assortment of applications on which it has been trialed. We are optimistic that MinION will be eventually morphed into a potent tool for the diagnosis of drug hypersensitivity.

## Author Contributions

All authors listed, have made substantial, direct and intellectual contribution to the work, and approved it for publication.

## Conflict of Interest Statement

The authors declare that the research was conducted in the absence of any commercial or financial relationships that could be construed as a potential conflict of interest.
